# Magnetothermal-based non-invasive focused magnetic stimulation for functional recovery in chronic stroke treatment

**DOI:** 10.1038/s41598-023-31979-w

**Published:** 2023-03-27

**Authors:** Hohyeon Kim, Jihye Kim, Jahae Kim, Seungjun Oh, Kangho Choi, Jungwon Yoon

**Affiliations:** 1 School of Integrated Technology, Gwangju Institute of Science and Technology, Gwangju, 61005 South Korea; 2 Department of Neurology, Chonnam National University Hospital and Medical School, 8 Hak-dong, Dong-gu, Gwangju, 501-757 South Korea; 3 Department of Nuclear Medicines, Chonnam National University Hospital and Medical School, 8 Hak-dong, Dong-gu, Gwangju, 501-757 South Korea

**Keywords:** Preclinical research, Stroke, Nanoparticles

## Abstract

Magnetic heat-based brain stimulation of specific lesions could promote the restoration of impaired motor function caused by chronic stroke. We delivered localized stimulation by nanoparticle-mediated heat generation within the targeted brain area via focused magnetic stimulation. The middle cerebral artery occlusion model was prepared, and functional recovery in the chronic-phase stroke rat model was demonstrated by the therapeutic application of focused magnetic stimulation. We observed a transient increase in blood–brain barrier permeability at the target site of < 4 mm and metabolic brain activation at the target lesion. After focused magnetic stimulation, the rotarod score increased by 390 ± 28% (p < 0.05) compared to the control group. Standardized uptake value in the focused magnetic stimulation group increased by 2063 ± 748% (p < 0.01) compared to the control group. Moreover, an increase by 24 ± 5% (p < 0.05) was observed in the sham group as well. Our results show that non-invasive focused magnetic stimulation can safely modulate BBB permeability and enhance neural activation for chronic-phase stroke treatment in the targeted deep brain area.

## Introduction

Ischemic stroke is a leading cause of death that induces varying levels of damage to brain function. Recovery from brain disability caused by stroke is one of the most challenging tasks in stroke research and clinical trials. While neurologic motor, sensory, and cognitive deficits are common after stroke, treatment is limited. For decades, various treatments have been proposed to alleviate the brain damage caused by stroke and to restore functional recovery.

Traditional strategies include pharmacological approaches using fluoxetine or amphetamine^1,2^, recovery using biomaterials^3,4^, transcranial stimulation^5–9^, optogenetic modulation^10–12^, ultrasound^13–18^, and robotic devices that induce changes in neural plasticity^19^. In addition, the clinical use of nanoparticle targeting is under investigation^20^. Stimulation using magnetic nanoparticles (MNPs) is generally non-invasive and elicits an immediate response^21,22^. Exposure of MNPs to a magnetic field releases heat through three independent mechanisms: Néel relaxation, Brownian relaxation, and hysteresis loss^23^.

Magnetic hyperthermia is an emerging technology that enables non-invasive brain stimulation via modulation of cortical excitability. Magnetic hyperthermia can activate transient receptor potential vanilloid 1 (TRPV1), which is widespread throughout the brain^24,25^, and is sufficient to modulate brain synaptic transmission and evoke neuronal firing in the motor cortex^21,26^. Thermal exposure by magnetic hyperthermia also leads to temporal disruption of BBB permeability^27^. Despite the clinical potential of magnetic hyperthermia, many studies have shown that hyperthermia in acute-phase stroke models leads to worse outcomes, because the penumbra is converted into an irreversible lesion^28–30^. However, the thermal effect on the chronic-phase after histological recovery has not been explored. In addition, focused magnetic heating could minimize undesired adverse effects and induce effective stimulation of the targeted area in chronic-phase stroke. Here, we applied focused magnetic heating to the target lesion to evaluate the impacts of functional neuromodulation and transient changes in BBB permeability to enhance therapeutic delivery in a chronic-stroke preclinical rat model. To our knowledge, investigations of focused hyperthermia in chronic stroke models have not been conducted.

## Results

### Physical properties of MNPs

Particle size, surface, and structure have important roles in hyperthermia applications^31–33^. To confirm that commercially available nanoparticles are suitable for in vivo experiments, we measured the efficacy of MNPs in a phantom environment. Microscopy shows the structural size distribution of 25 ± 3 nm MNPs, measured using transmission electron microscopy (Supplementary Fig. 1a). The average hydrodynamic size of particles moving in blood vessels was 45 ± 15 nm (Supplementary Fig. 1b). Magnetization, an important indicator of the effects of hyperthermia, was used to evaluate suitability for in vivo experiments according to the maximum magnetization value and slope of the hysteresis loop^34^. The saturation magnetization value of MNPs measured in the liquid state was 67 emu/g (Supplementary Fig. 1c), indicating a high magnetic moment for in vivo experiments.

The relaxation that dominates the heat generation varies according to the degree of freedom of the particles. Therefore, the influence of two types of relaxations, which may vary depending on the experimental conditions, was evaluated. Magnetic heating experiments of liquid particles showed that the heating efficiency was higher at higher frequencies than at lower frequencies (Supplementary Fig. 2a). This demonstrates the high efficiency of Néel and Brownian relaxation at high frequencies in the absence of relaxation constraints. Both relaxations are highly efficient in the fluid state, where the particles are free to move. The increase in temperature of the nanoparticles inside the phantom (Supplementary Fig. 2b) indicates that the efficiency is also higher at higher frequencies, even with limited Brownian relaxation. When the particle penetrates the tissue and becomes immobilized or highly viscous, the Brownian relaxation weakens, and the Néel relaxation becomes dominant. However, the results of the liquid particle and phantom experiments show that both relaxations show high efficiency at high frequencies, which can compensate for the heat generation lost by the medium.

### Focused magnetic heating

The magnetic flux density for each distance of the gradient was simulated through COMSOL software based on the measured magnetic field. When the measured gradient field was 2.1 T/m, a magnetic flux of 7 mT at 5 mm from the center was recorded. In the simulation, the FFP generated according to the actual field strength was estimated to be a 1-cm-diameter sphere. At ≥ 5 mm from the center, the heating effect was lost because the gradient field was stronger than the magnetic field of the coil; heating efficiency increased as it approached the center. The theoretically expected size of the FFP can be calculated by the magnetic field H and gradient field G.

1 $$\frac{{d}_{FFP}}{2}=\frac{H}{G}$$

**Figure 1:**
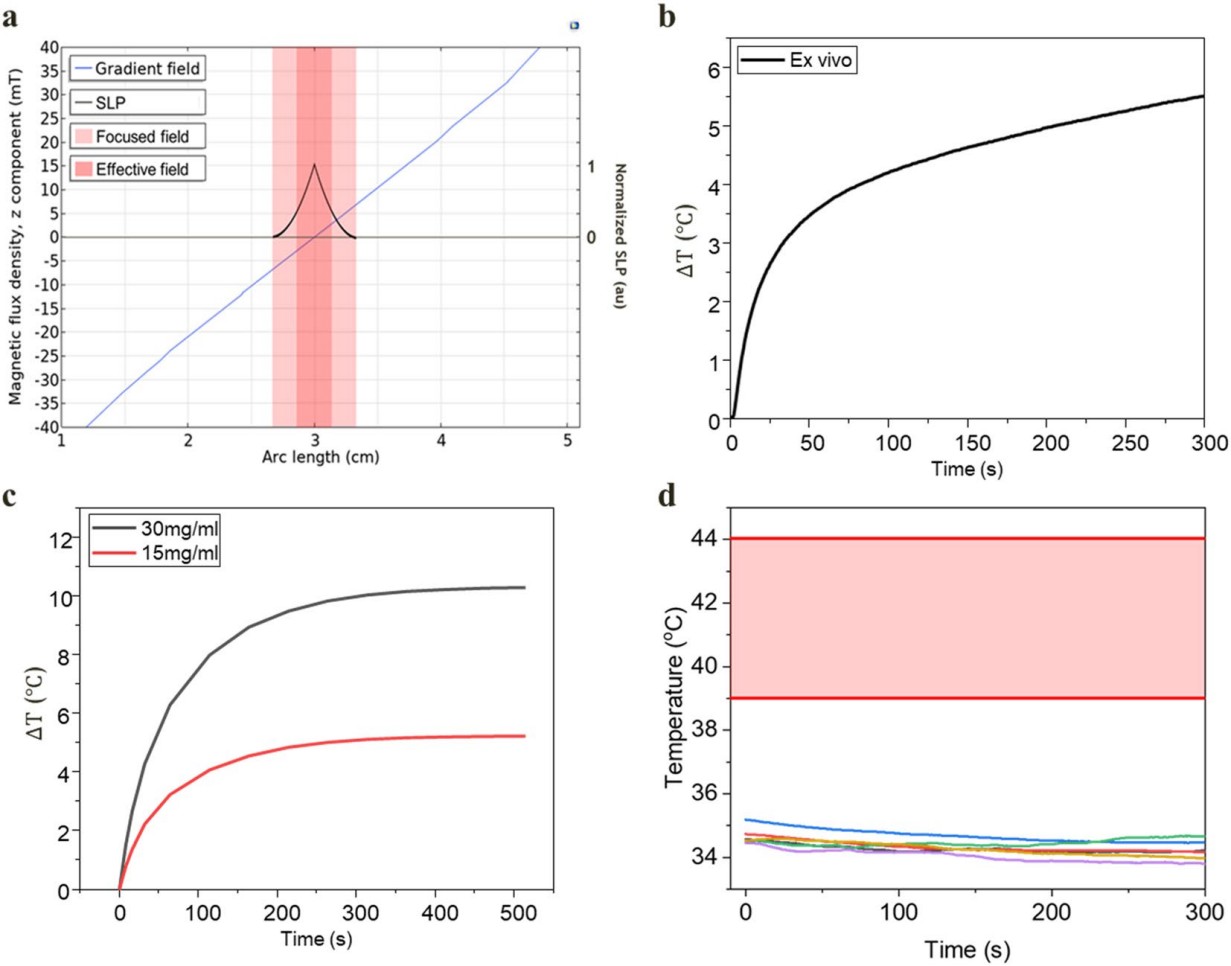
Simulation and experimental results of temperature control. (**a**) FFP area, with the generated gradient field and SLP inside the area. The FFP is generated up to a distance where the gradient field and external field strength match, which is equivalent to the theoretical calculated distance of 7 mm. The theoretically expected size of the FFP can be calculated by the magnetic field H and gradient field G; $\frac{{d}_{FFP}}{2}=\frac{H}{G}$. The magnetic heating that begins at the boundary becomes stronger as it approaches the center ($SLP\propto {H}^{2}$). An effective FFP can be expected to generate heat up to 3.5 mm in diameter, which is approximately half of its maximum size. (**b**) Ex vivo temperature increase with 15 mg/mL concentration. (**c**) Simulation results of temperature increase in vivo using different concentrations. (**d**) The brain baseline temperature before magnetic stimulation was maintained at 34 °C (n = 6), which was slightly lowered by anesthesia. The range of 39°–44° was set for the experiment to induce stimulation and change of BBB permeability.

Figure 1a shows simulation results of the gradient field, region of focused heating, and normalized SLP inside the region. The theoretical maximum size of the FFP is 7.3 mm; the range directly affected by stimulation is regarded as 3–4 mm based on the temperature increase or heating efficiency for the application. Alternating magnetic fields are generated at the point where the magnetic field of the induction coil and the flux of the gradient field are matched; close to the center, the SLP value increases in proportion to the square of the field strength H. Based on the simulation results, it is possible to designate a range of 4 mm that generates sufficient heat generation efficiency under the influence of an alternating magnetic field.

The temperature of each sample distributed inside the phantom holder (Supplementary Fig. 2c) was measured using an infrared camera. The temperature increased by > 9 °C in the center corresponding to the FFP area; the region outside the FFP area was maintained at room temperature. A temperature difference between the target point and the region outside the FFP area was > 30 °C (Supplementary Fig. 2d), according to direct measurements with the probe. The temperature distribution measured by the IR camera in the phantom holder confirms the increase in temperature by the FFP (Supplementary Fig. 2c). Therefore, by adjusting the gradient magnetic field and magnetic strength from the coil, the desired position of the FFP can be adjusted, along with the ROI to which the magnetic field will be applied. Based on the FFP, hyperthermia can be induced only in the desired region, even in an environment where particles are scattered.

An experimentally determined SLP value of 139 w/g was used in vivo simulation model. Based on SLP in vivo temperature prediction calculated by FFP heating, the simulation result of temperature increased by 10° was verified when a concentration of 30 mg/mL was applied. Moreover, a temperature increase by 5° for safe and continuous stimulation was verified when the concentration of 15 mg/mL was applied (Fig. 1c). Since the baseline temperature of the rat brain was 34 degrees (Fig. 1d), the target temperature based on measurement was set to the maximum range of 44° for stimulation^35,36^ within the safe range and the minimum range of 39° for increasing BBB permeability^37^. As a result of measuring the temperature increase in an ex vivo environment using the optimized parameters, a temperature increase of 5.5° was confirmed without the blood flow effect (Fig. 1b). Since the heating efficiency of nanoparticles is determined by the strength and frequency of the magnetic field applied to the characterized particle properties, ex vivo temperature measurement ensures the consistency of in vivo temperature rise.

### Analysis of IVIS imaging and immunohistochemistry

**Figure 2:**
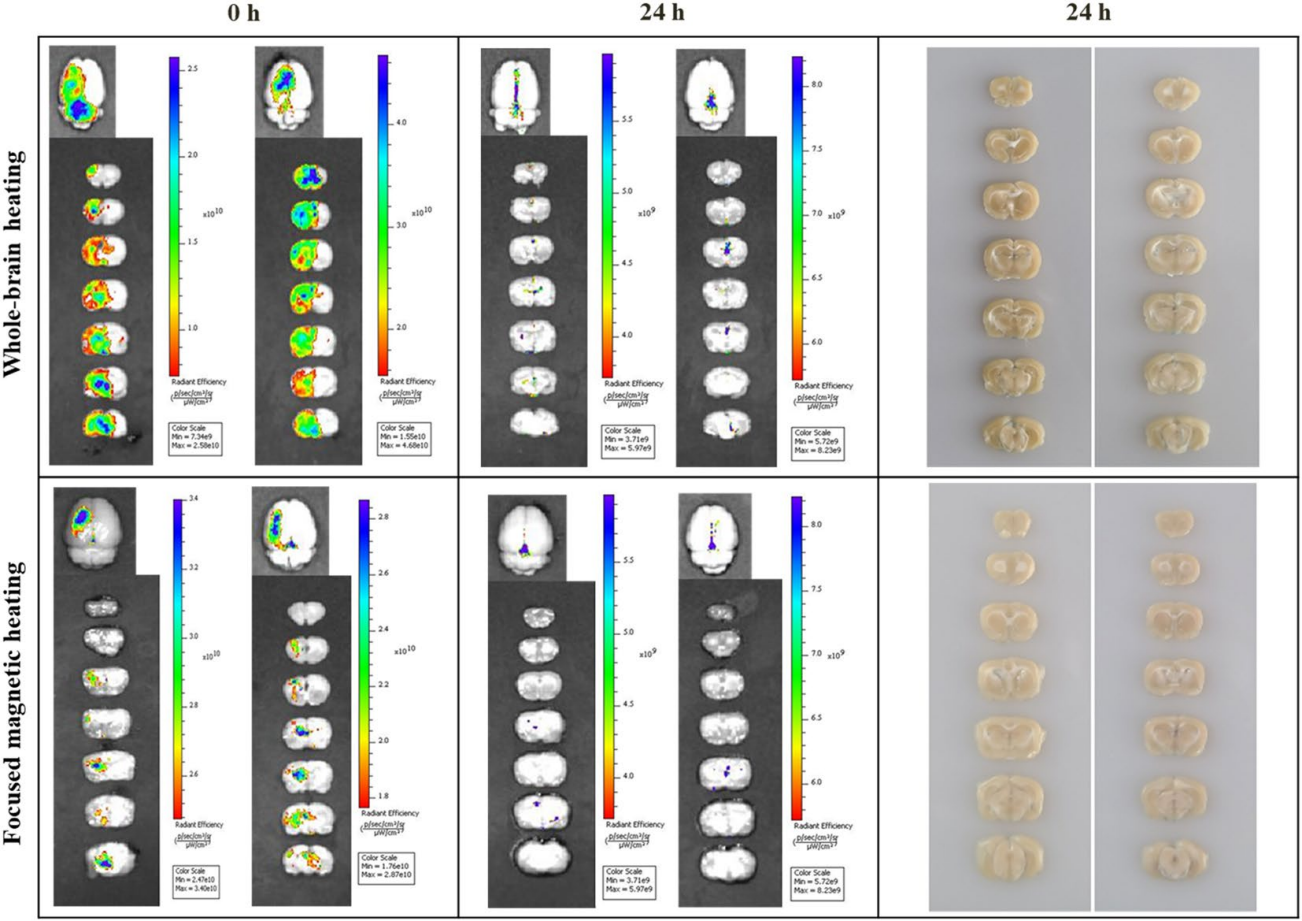
Evans blue fluorescence. Each row shows the recovery of BBB permeability after whole-brain (n = 10) and focused heating (n = 10) in a normal rat. Each column shows BBB recovery immediately after focused heating and after 24 h, as well as a brain-slice image after 24 h. The IVIS image showed that the permeability does not change with a magnetic field alone in the right cerebral hemisphere where the particles are not spread. Only by the MNP to which the magnetic field was applied could affect the area, even in the left hemisphere region where the particles are spread, the BBB permeability change occurred only in that region where a focused magnetic field was applied.

Stimulation-induced changes in BBB permeability in the left hemisphere were evaluated using Evans blue staining. To determine the duration and recovery of magnetic heating-induced changes in permeability, Evans blue dye was injected after heating, and at 24 h and 48 h after heating. The animals were separated into three groups depending on the animal model and the region in which the magnetic field was applied. Brains were collected from each group and Evans blue fluorescence was analyzed. Figure 2 shows changes in the BBB over time for each heating method, along with an image of a brain slice after 24 h.

IVIS imaging of whole-brain heating in normal rats was performed daily. Evans blue staining was widespread and uniformly measured at the region where the particles were released. In the left hemisphere, the dye was uniformly spread throughout the tissue. Fluorescence measurement at 24 h after heating showed that the BBB was almost recovered at all sites; almost no dye remained. After 48 h, there was no difference between the heating and control groups. Thus, stimulation applied to the whole-brain after particle injection can transiently increase BBB permeability and recovery within 24 h, without causing tissue destruction.

IVIS imaging using focused magnetic heating at the desired site produced results that were distinct from whole-brain heating. Immediately after heating, Evans blue fluorescence was higher in the striatum, which was the site that received the 4-mm focused field. If the FFP area size is insufficient, the stimulated site may be invalid; if the FFP area size is excessive, targeting may be difficult and side effects may occur in surrounding tissues. The effective focused heating area of 4 mm induced in FFP was sufficient to stimulate the lesion site in the stroke model. Indeed, Evans blue fluorescence was strongest in the area where the FFP was applied; it decreased with increasing radial distance from the FFP area. Similar to the whole-brain group, BBB recovery was observed within 24 h in the focused magnetic heating group. In normal rats, both heating methods caused a transient change in BBB permeability and recovery within 24 h.

**Figure 3:**
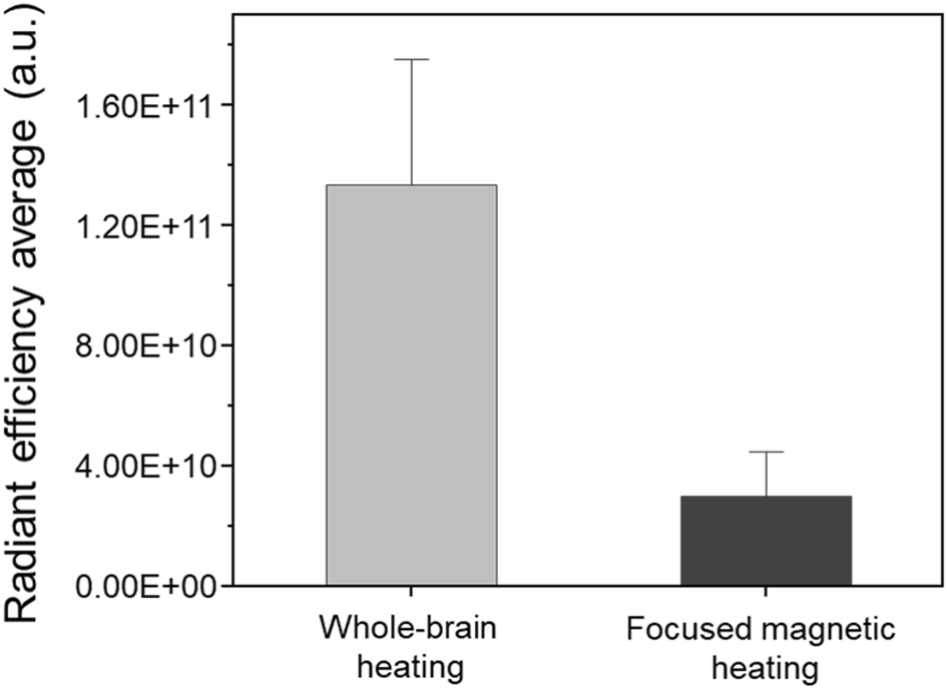
Average IVIS fluorescence value within ROI. Evans blue leakage inside the ROIs of the whole-brain (n = 10) and focused heating (n = 10) group were quantitatively evaluated. IVIS fluorescence by Evans blue leak to which a focused magnetic field was applied showed a more localized area than when the magnetic field was applied to the entire area.

In addition to the IVIS image, the group average fluorescence was quantitatively evaluated by comparing the whole-brain area and the focused magnetic area inside the ROI (Fig. 3). The radiant efficiency expressed as (p/s/cm^2^/sr)/(µW/cm^2^) was 1.33^11^ ± 2.58^10^ in the whole-brain heating group and 2.99^10^ ± 1.01^10^ in the focused magnetic heating group. The amount of leakage by individuals within the same group was similar, but the comparison by groups showed a significant difference (p < 0.001), and as a result, it was shown that localized heating delivered thermal energy only to a desired local area where focused magnetic heating was applied.

**Figure 4:**
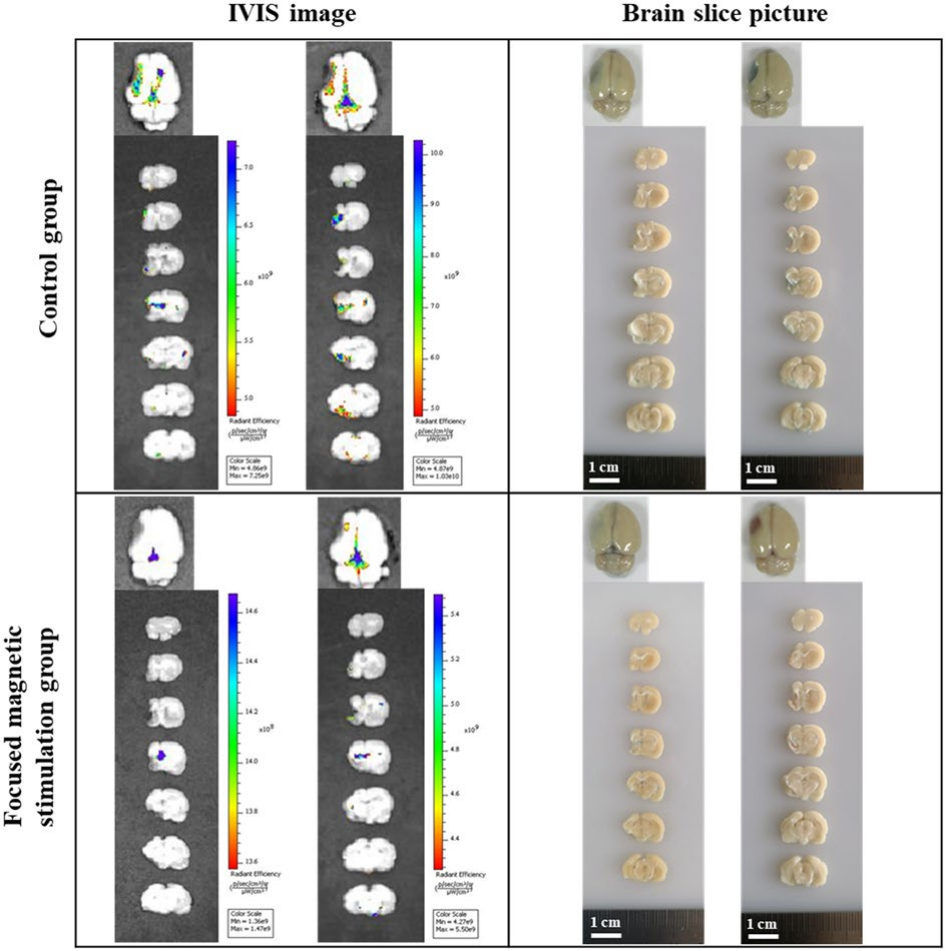
IVIS image and slice picture of stroke model. Brain damage due to stroke shows irreversible histological damage. Therefore, focused heating induces functional recovery by stimulating the remaining tissue around the lesion. By applying focused heating to the stroke model (n = 6), additional damage to the lesion while inducing nerve stimulation was evaluated. In stroke models, the BBB was destroyed as a symptom of the stroke; although some Evans blue fluorescence was observed after 48 h, magnetic stimulation did not result in further deterioration. In all groups, the transient increase in BBB permeability induced by hyperthermia was safely recovered over time.

In stroke models, the BBB is destroyed as a symptom of the stroke; while some Evans blue fluorescence was observed after 48 h, further destruction of the BBB by magnetic heating was not observed (Fig. 4). In all groups, the transient increase in BBB permeability was safely recovered after stimulation.

**Figure 5:**
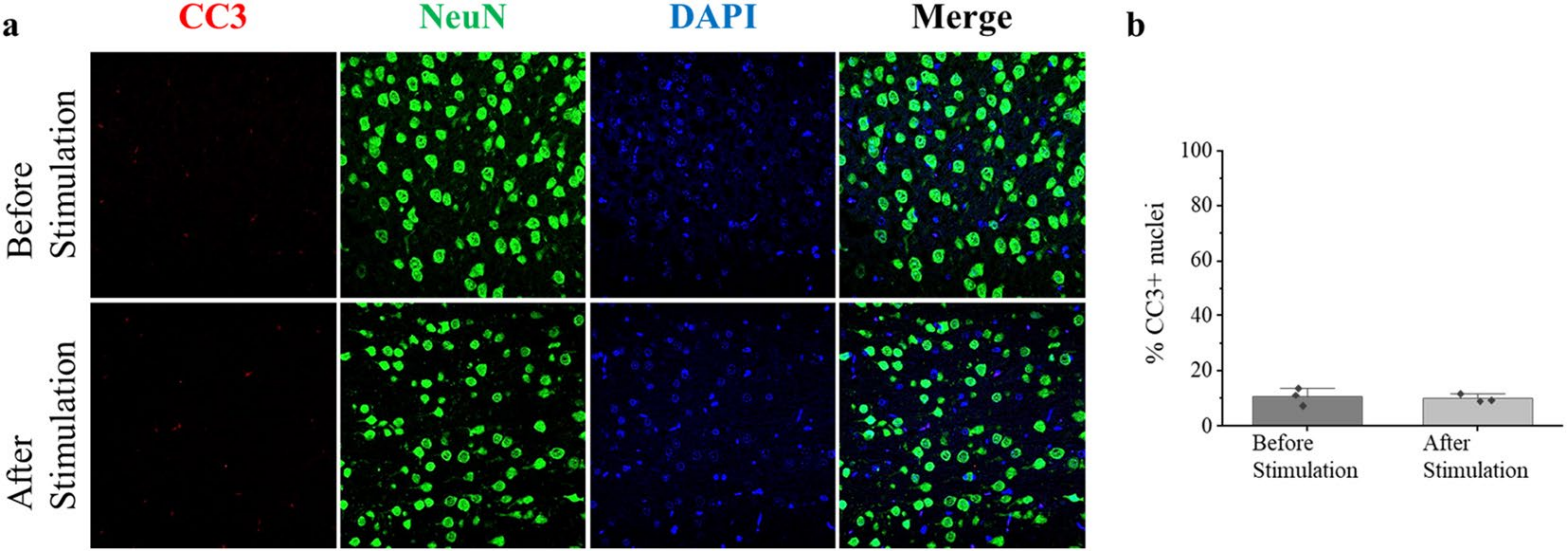
Immunohistochemistry staining before and after stimulation of the sham group. Original magnification, $\times$200. (**a**) Each of the images is representative of a field of immuno-stained cells. Each merged image includes the nuclear marker DAPI, CC3, and NeuN. Damage by the surgical process before stimulation and damage by the stimulation protocol was not confirmed. No significant changes such as cell damage indicating apoptosis or morphological changes of neuronal nuclei were observed in CC3 and NeuN. (**b**) The bar charts show the percentage of CC3-positive cells relative to the percentage of DAPI stained nuclei, in cells before and after stimulation. Each data point represents a biological replicate (n = 3).

These results show that BBB permeability was increased by magnetic stimulation. The issue in nanoparticle-based in vivo hyperthermia experiments is that the heating efficiency is very low due to the immobilized state of the particles in the tissue or the loss of heat by blood flow. Therefore, leakage of Evans blue from tissue slice allows a qualitative assessment that the particles can generate thermal energy that raises the temperature to 39° in vivo. The increase in BBB permeability indicated that sufficient thermal energy was delivered to the affected area; a stimulatory effect was delivered to the tissue. IVIS imaging revealed no leakage of Evans blue from unintended areas in the stroke model or normal rats. Moreover, in subsequent behavioral tests, stimulation by hyperthermia promoted function and did not disrupt tissue. Focused magnetic heating can minimize undesired effects on normal tissues outside stroke sites. Indeed, as a result of immunohistochemistry in the sham group (Fig. 5), it was confirmed that there was no difference in cleaved caspase-3 (CC3), a marker of apoptosis, between rats sacrificed before and after stimulation. Tissue damage such as apoptosis was not induced by stimulation (Supplementary Fig. 3), and the number or morphology of neuronal nuclei (NeuN), which are a marker of neurons, does not change, proving the safety of stimulation. Thus, focused magnetic heating can be used clinically to control treatment areas and transient changes in BBB permeability.

### Behavioral evaluation and neurologic deficit score

Behavioral evaluation using the rotarod test was used to assess the stimulation caused by focused magnetic heating. NDS before and after focused magnetic heating treatment were compared in the control, sham, and focused magnetic heating groups.

**Figure 6:**
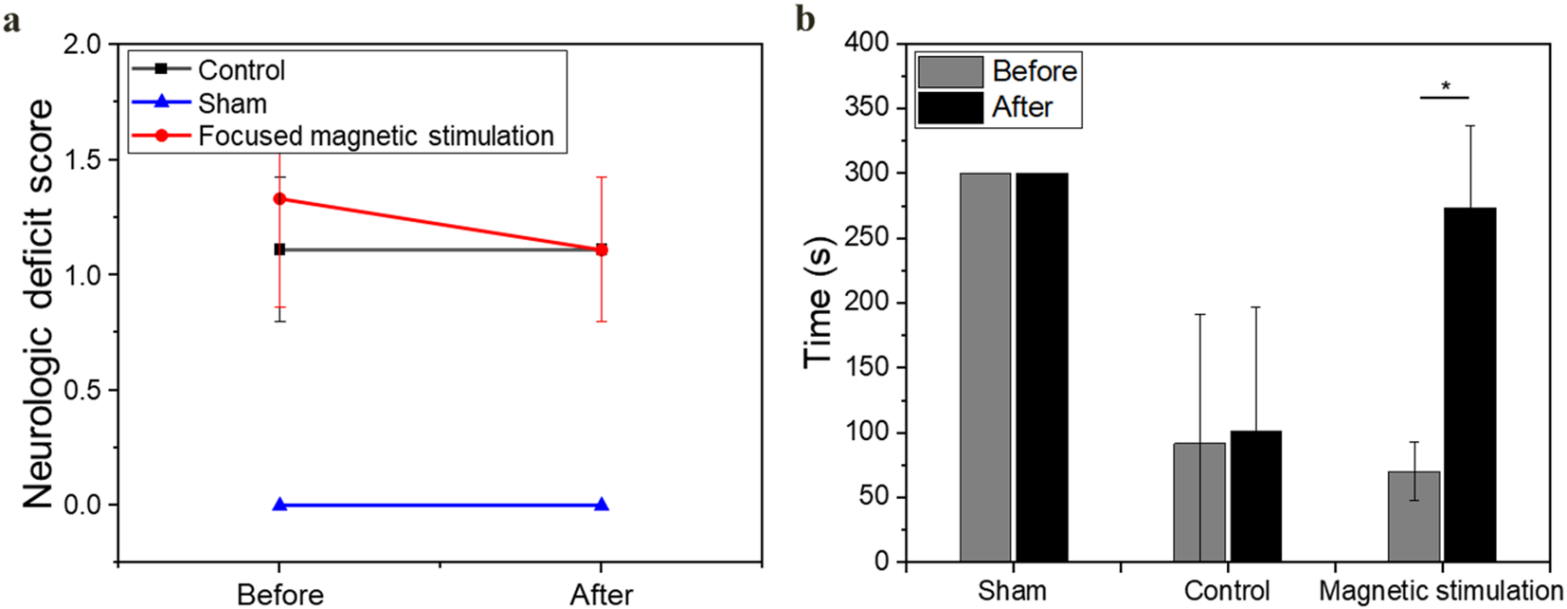
Behavioral assessment analysis and NDS for each group. (**a**) No deficit in the sham group (n = 9) was observed. The NDS value of the control group (n = 6) was maintained at 1 point. The NDS in the magnetic stimulation group (n = 9) was recovered after stimulation. (**b**) No decline or recovery of motor function was observed in the normal model of the sham group without stroke. In the control group, a stroke-induced decline in function was identified and did not recover over time. The magnetic stimulation group showed significant motor function recovery after stimulation (p < 0.05).

In the control group, which underwent MCAO stroke without heating, the NDS did not change after 1 week; the average was maintained at 1.11 ± 0.31 point (Fig. 6a). Recovery of motor function was not observed over time after stroke-induced impairment of motor function. In the control group, neither NDS nor behavioral evaluation recovery were observed before or after the experimental period.

No deterioration in motor function was observed at the normal rat in sham group after focused magnetic heating. All behavior-evaluation results were recorded 300 s before heating; no difference was observed. The NDS was maintained at zero; no deficit was found. In sham group, no functional decline or recovery in terms of behavioral evaluation or NDS was observed before or after the experiment.

After the injection of particles into the stroke model, focused magnetic heating of the target area was performed in the experimental group; functional recovery was evaluated. Before heating, a significant stroke-related decrease in motor function was observed in the focused magnetic stimulation group. The average NDS was 1.33 ± 0.47 points, and the time spent in the rotarod test was 70.1 ± 22.8 s (around one-third of the time in the sham group).

After application of focused magnetic heating to the stroke area for 6 days, the NDS improved by approximately 15%, from 1.33 ± 0.47 to 1.11 ± 0.31, however, it was not significant. The behavioral evaluation (Fig. 6b) revealed remarkable restoration of motor function after focused magnetic heating, from 70.1 ± 22.8 to 273.5 ± 62.8 s; this motor ability was similar to the sham group. A significant difference was observed after heating (p < 0.05). To our knowledge, these are the first experimental results to confirm restoration of motor function by focused magnetic hyperthermia after chronic stroke. The recovery of motor function by stimulation will be an important starting point in studies of stroke and recovery mechanisms.

### Evaluation of metabolic activation in the PET/CT brain image

Several PET/CT images and numerical analyses of SUV were performed in the stroke-model rats to evaluate the metabolic response of the brain to thermal stimulation. To explain the cortical metabolic response in terms of motor function recovery, activation at the target site was verified by ^18^F-FDG micro-PET imaging.

**Figure 7:**
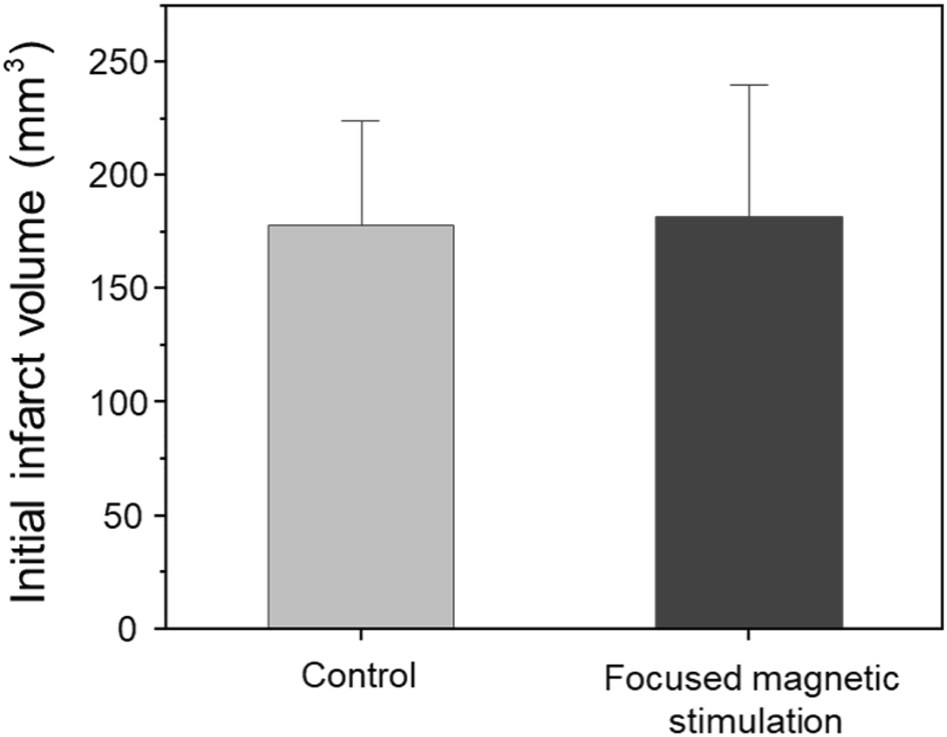
Initial infarct volume of stroke model prepared by MCAO method. The initial infarct size of stroke models prepared by the MCAO method was compared. The prepared stroke model (n = 15) was divided into a control group to which no magnetic heating was applied and a group to which focused magnetic heating was applied. Initial infarct size between the two groups showed no significant difference.

In order to analyze the brain PET image, the volume of the initially generated infarct acquired from micro-CT was quantitatively evaluated (Fig. 7). Each stroke model was compared by group, and the initial infarct volume of the control group was 177.87 ± 42.1 mm^3^ and the initial infarct volume of the focused magnetic stimulation group was 181.58 ± 40.6 mm^3^. The p-value was 0.931, showing no significant difference between the two groups. Consistency of initial infarcts indicates that brain activation was induced by the focused magnetic stimulation.

**Figure 8:**
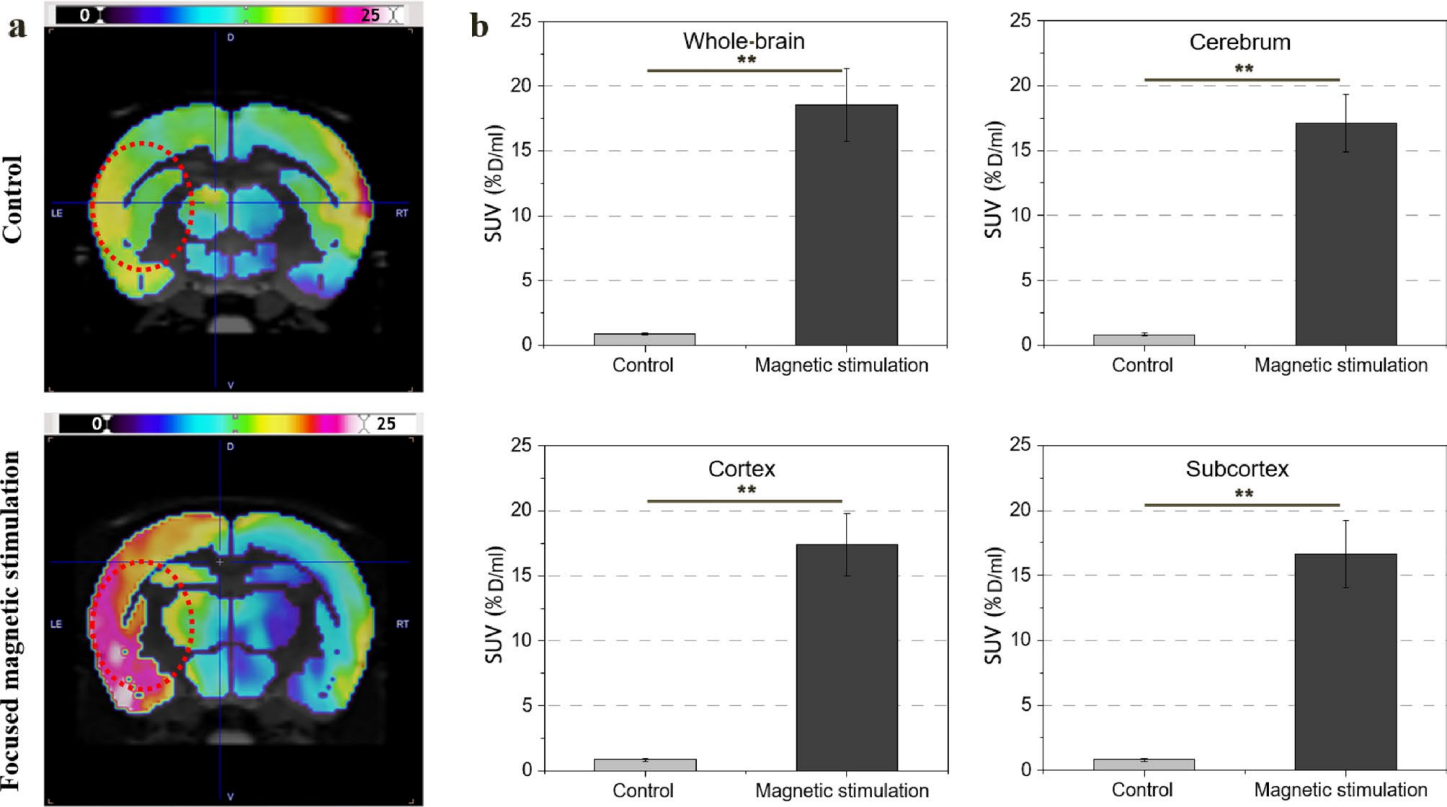
Changes in SUV index in the control (n = 6) and focused magnetic stimulation groups (n = 9). (**a**) PET/CT images of brain activation after heating in the control and focused magnetic stimulation groups. (**b**) Comparison of SUV index in the cerebrum, cortex, and subcortex, and whole-brain average with and without magnetic stimulation. In the group to which the focused magnetic field was applied to the stroke lesion, a significant increase in metabolic activity was observed (p < 0.005).

Figure 8a shows the difference between the control group and the focused magnetic stimulation group with left-hemisphere stroke. The closer the green at the bar center is to the black, the more metabolism decreases, and the closer it is to the red, the more it increases. In the MCAO model, a significant difference in activation at the target site was observed between the group that did not receive hyperthermia and the group that received focused magnetic hyperthermia for 6 days. In the control group unaffected by hyperthermia, there was almost no change in brain metabolic activity after 6 days. SUV index of the whole-brain, cerebrum, cortex, and subcortex in the control group were 0.9 ± 0.1, 0.83 ± 0.09, 0.85 ± 0.1, and 0.81 ± 0.09, respectively. In contrast, metabolism at the target site was increased in the focused magnetic stimulation group that underwent focused magnetic heating in the left brain. SUV index of the whole-brain, cerebrum, cortex, and subcortex in the focused magnetic stimulation group were 18.57 ± 2.8, 17.1 ± 2.2, 17.42 ± 2.4, and 16.66 ± 2.6, respectively. Overall metabolic function was increased at the stroke site; PET imaging revealed significantly increased metabolic activity in the somatosensory and motor cortices of the cerebral cortex of rats that had been subjected to a focused magnetic field. Figure 8b shows that the SUV indices in the cerebrum, cortex, and subcortex of the hemisphere affected by focused stimulation were significantly increased, compared to those values of the control group. The overall average SUV was approximately 20-fold larger in the stroke area of the group that had undergone focused magnetic field application.

**Figure 9:**
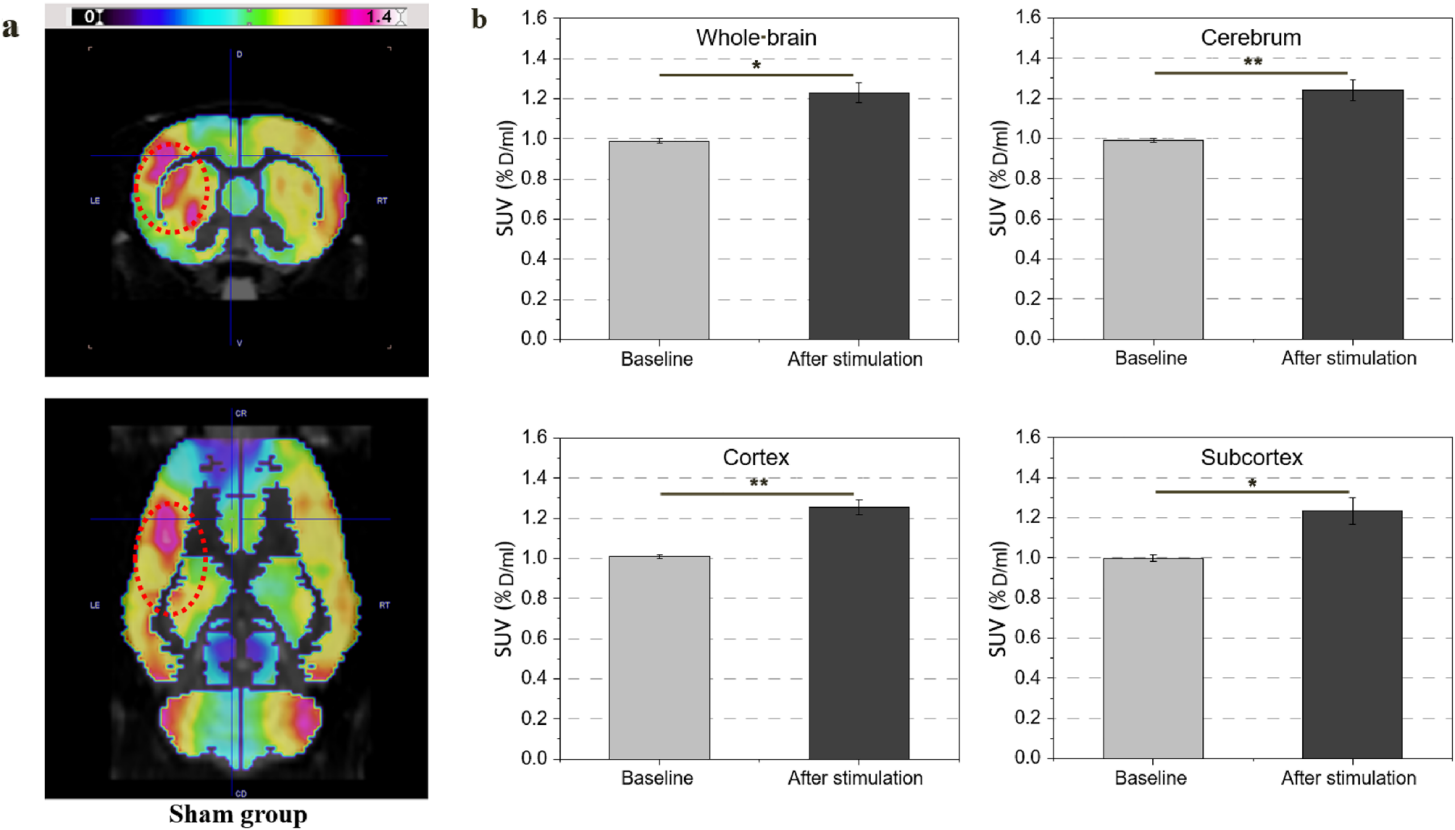
Changes in SUV index in the sham group (n = 9). (**a**) PET/CT images of brain activation before and after heating in the sham group. (**b**) SUV index at baseline and after heating in the cerebrum, cortex, and subcortex, and whole-brain average (p < 0.05). Increased metabolic activation of 24% was observed even in the same brain site without stroke.

Figure 9a shows the difference between the baseline before heating and the activation after heating in the sham group. A mild stimulation effect was observed at target site in the sham group. After stimulation, SUV index of the whole-brain, cerebrum, cortex, and subcortex of normal rat in the sham group were 1.23 ± 0.05, 1.24 ± 0.05, 1.25 ± 0.03, and 1.23 ± 0.06, respectively. Figure 9b shows the SUV index of the activated region. Compared with the baseline, a 24% increase in metabolism was observed after heating. We found focused magnetic heating was equally capable of stimulating intact brain regions.

The difference between the experimental group and the control group was significant (p < 0.005), and the comparison between the before and after stimulation of the sham group also showed a significant difference (p < 0.05), as determined by the nonparametric Mann–Whitney U test. All of these statistical significances were below a p-value of 0.05. Our results show that the effects of hyperthermia in the chronic phase were distinct from the heat-induced exacerbation that occurred in the acute phase^28–30^. Behavioral assessment, NDSs, and brain activation showed improvement after hyperthermia. The discovery that hyperthermia can induce motor function recovery may lead to improved treatment protocols and rehabilitation strategies according to the pathologies of diseases associated with movement disorders.

## Discussion

Our study showed that by adjusting the magnetic strength of the gradient field, the desired position of the FFP can be adjusted, along with the ROI to which the magnetic field will be applied. We showed that FFP-based stimulation can safely modulate BBB permeability and activate metabolic activity in target brain regions by inducing stimulation only in desired areas, even in a particle-scattered environment. Simulation results and an increase in tissue temperature showed that a concentration of 30 mg/mL could induce a temperature rise up to 44° from the baseline temperature, leading to BBB opening and metabolic activation^36^. IVIS imaging revealed no leakage of Evans blue from unintended areas in the stroke model or normal rats and showed spontaneous recovery of BBB permeability after 24 h. Although this transient change allows targeted heat transfer, extensive/intense stimulation with sufficient strength to disrupt tight junction increases the risk of irreversible damage. In addition, the application of a wide range of magnetic fields suggests that the heat generated by the particles can transmit stimulation to undesired areas; focused mild stimulation is a reasonable alternative for neuromodulation. Therefore, 39°^37^, the temperature at which the BBB begins to open, was chosen as the target temperature for stimulation. By using a concentration of 15 mg/mL, the saturation temperature increased by 5°, and the temperature was confirmed both by simulation and ex vivo experiments. The increase in BBB permeability indicated that sufficient thermal energy for stimulation was delivered to the focused area. Neuromodulation and behavioral changes accompanying such BBB opening have been observed in recent studies^38,39^. We confirmed through quantitative analysis of average fluorescence of groups in ROI that localized BBB opening can be achieved through focused magnetic heating. We confirmed motor function recovery and brain metabolic activation by targeted neuromodulation through behavioral evaluation and PET/CT results in the MCAO model. In the group receiving targeted stimulation treatment, motor function based on the rotarod test increased nearly 4 times, and metabolic brain activation increased 20 times. Initial infarction, metabolic increase, and recovery of motor function were all evaluated quantitatively, and the consistency of initial infarction between groups indicates that metabolic increase and functional recovery are due to focused magnetic stimulation.

To date, few studies have investigated hyperthermia for the treatment of chronic stroke. Most recovery from stroke impairment occurs in the first 1–3 months as a result of spontaneous biological recovery and increased responsiveness to training^40^. The effectiveness of recovery-inducing motor training reduces after a sensitive period (1 month in rodents). Our findings suggest that focused stimulation of the lesion may increase neuroplasticity in chronic phase stroke when the effect of exercise training is difficult to expect because it has exceeded the sensitive period. Indeed, evidence has been provided that recovery of motor function after stroke is associated with increased brain plasticity^41^. Our findings imply that focused stimulation of the stroke lesion can restore motor function and increase functional plasticity for the treatment of chronic stroke. The changes in BBB permeability and brain metabolic activation observed in this study imply that focused magnetic hyperthermia can be used universally in chronic stroke treatment.

We have demonstrated that thermal stimulation can induce behavioral and neural responses, but the exact mechanism is still unclear. The activation of ion channels can explain neuronal activation by focused magnetic stimulation. Thermal stimulation can activate Ca^2+^ permeable transient receptor potentials (TRPV1-4 and TRPM2), members of the TRP superfamily^42^. One central mechanism of thermal stimulation can be the cation channel response of heat-sensitive TRPV1^21,22,27,43,44^. Some authors have injected adeno-associated virus serotype 5 (AAV5) to achieve overexpression of TRPV1^21,22^. In this study, the natural expression of TRPV1 was induced without virus injection. The temperature increase above a threshold opens TRP channels, and depolarization occurs in dorsal root ganglion neurons. The Ca^2+^ influx by depolarization is sufficient to trigger neuronal activity, releasing neurotransmitters into the synapse and firing an action potential. Stimulation at temperatures lower than the noxious heat threshold can also depolarize TRPV1 hippocampal neurons and open approximately 20% of channels^45^. Because TRPV1 has high single-channel conductance, calcium influx by depolarization is sufficient to trigger neuronal activity^46^. Stable and sustained neuronal activation has been reported even with temperature rises to 41 °C and 46 °C^47^. The authors noted no significant difference in the activation of the somatosensory area between the 41° and 46° of stimulation, suggesting that stimulation is possible at temperatures lower than the noxious heat threshold. Furthermore, the recovery of motor function and increase in brain metabolism shown in our experiment imply that ion channels can be activated even near 39° induced by the conditions of 595.4 kHz and 7 mT. In addition, above studies showed that heating caused depolarization in all neurons, not just heat-sensitive neurons. Therefore, our results suggest the experimental possibility that sufficient neuronal activation can be achieved by focused magnetic thermal stimulation by activating ion channels^21^.

This activation of ion channels can lead to neuroplasticity for the functional restoration of the brain. Neurons surviving around the lesion after stroke contributes to active recovery through adaptive circuit plasticity. The hypothesis of an endogenous mechanism to partially restore the lost function after stroke is that survived neurons from injury are recruited to take over the roles of dead neurons and undergo synaptic remodeling^48–50^. Synaptic remodeling triggers synaptic plasticity by regulating the rewiring and strengthening of neural circuits with the stimulation of astrocytes^51^. Calcium manipulation by repeated stimuli of astrocytes activates NMDA receptors, and the release of gliotransmitters by inhibitory or excitatory synaptic transmission consequently induces long-term potentiation for functional recovery^52–56^. The proposed focused magnetic heating can stimulate astrocytes and lead to action potentials by modulating excitability closer to the threshold of depolarization^57^. The excitability by stimulation can increase the recovery by activating neuronal responses to induce neuroplasticity and assisting rehabilitation in restoring impaired motor functions^58^. Our results of functional recovery suggest that magnetic stimulation can potentially contribute to the healing of damaged neurons from ischemic injury and amplify endogenous brain repair mechanisms by neurogenesis, angiogenesis, immune modulation, and promoting synaptic remodeling, including increased neuronal plasticity.

Several advantages can be cited for the use of focused magnetic stimulation techniques for clinical practice. The mechanical configuration for focused magnetic stimulation is relatively simple, which is a great advantage for extending to human-level systems, and the iron oxide particles for thermal stimulation can have high biocompatibility by dextran shell coating. In case of the work^27^ injected in the same way as in this experiment shows that particles are accumulated in blood vessels, suggesting that repeated thermal stimulation is possible with these accumulated particles. Nanoparticles inside the body are cleared by Kupffer cells, endothelial cells, and B cells in the liver, or are filtered by the glomeruli in the kidney^59,60^. MNPs administered in this way are removed from the bloodstream over time. Therefore, the conceptual configuration has the advantage for clinical use. The technique of focusing a magnetic field on the desired area (< 4 mm) through a gradient magnetic field can induce local neuro-stimulation with high precision on a human scale. Our technological development for magnetic stimulation allows all brain regions, including deep brain regions, to be targeted in a non-invasive manner. Focused magnetic stimulation also has advantages over conventional therapeutic studies such as ultrasound and transcranial magnetic stimulation (TMS). Although there is evidence that the application of focused heating by ultrasound can increase angiogenesis, promote tissue healing, and increase neurotrophic factor levels at the target site^16,17^, ultrasound cannot penetrate bone or cavities. This limitation restricts the use of ultrasound for major organs (such as the heart and brain). While TMS can safely stimulate the target site in a non-invasive manner, its depth of penetration is limited to the superficial area. While MNP-based stimulation cannot induce action potential firing through direct current induction due to a magnetic field 100 times weaker than that of TMS, focused magnetic heating involves direct thermal stimulation mediated by nanoparticles and can target sites at any depth or location. Therefore, focused magnetic stimulation, which can target deep areas while retaining the advantages of conventional TMS and ultrasound, could be a viable approach to reaching areas inaccessible through conventional ways.

In conclusion, we demonstrated that non-invasive focused magnetic stimulation can minimize undesirable effects on normal tissues outside the stroke lesion and activate neuronal recovery in the targeted brain area for chronic stroke treatment. Contrary to the detrimental effects of thermal stimulation in the acute and subacute phases, thermal stimulation in the chronic phase was effective and showed that brain stimulation based on focused magnetic heating could activate neural circuits to restore motor function in a chronic stroke rat model. Our method may provide a safe neuromodulation treatment strategy for deep brain regions without surgery. However, several limitations must be overcome to apply the comprehensive strategy to clinical practice. Accurate temperature monitoring in vivo is required to quantitatively evaluate the effect of temperature. Since the heat generated by nanoparticles is highly localized depending on the distribution of the particles, direct temperature measurement with a probe is not a proper way for a clinic. Therefore, MR-based temperature distribution measurement that can confirm the temperature of the lesion site without contact can be an alternative^61^. Since the recovery of stroke motor function in the chronic phase using magnetothermal treatment has not been conducted before this study, persistent recovery should be addressed. In this study, since rehabilitation exercise was not given, the improvement of metabolism and improvement of the functional outcome can be judged entirely by the effect of focused magnetic stimulation. These results show that a better prognosis can be expected if the existing treatment, including rehabilitation exercise, which is known to be helpful for recovery after stroke, is maintained and additional magnetic stimulation is performed. TRP channel activation by temperature change and neuronal activation by ion channel regulation may activate an alternative circuit in lesions, and electrophysiological analysis is also needed to clarify the relationship between this plasticity and stimulation. In addition, the correlation of lesion location versus metabolic parameters and behaviors can provide insight into the mapping of functional localization in brain regions with future work. While identification of tissue biomarkers and the underlying mechanisms of neuronal changes require further experimentation, neuromodulation of transient changes in deep brain region BBB permeability and functional recovery through focused magnetic stimulation may serve as a promising research area for the treatment of chronic stroke.

## Materials and methods

### Characterization of MNPs

MNPs with a maghemite core covered by a dextran shell, also known as Synomag-D, were provided by Micromod GmbH (Rostock, Germany)^62^. A suspension diluted with distilled water was placed on a carbon Transmission electron microscope (TEM) grid and air-dried. TEM images of particles were acquired using JEOL JEM-2100 with a 200-kV electron beam. The hydrodynamic size of MNPs was measured by dynamic light scattering using Malvern Instruments Zetasizer Nano ZSP after 15 min of sonication. The magnetic property and hysteresis loop were measured using Lake Shore Cryotronics 7404-S VSM at room temperature. For evaluation of specific loss of power (SLP) of particles, the heating efficiencies of liquid particles were measured under high- and low-frequency conditions.

### Generation of focused magnetic field using field free point

An alternating square wave magnetic field was generated by a 17 turns coil with a diameter of 5 cm; to maintain room temperature, water was circulated inside the coil using a Grant LT ecocool 150. A pair of permanent magnets generates a gradient field on both sides to compensate high-frequency magnetic field in the rest of the area except for the central part. This central area created field-free point (FFP) concentrates the radio frequency field only in a local area. The magnetic field for stimulation is focused on the stroke lesion, and hyperthermia is applied. The focused magnetic field was applied over the targeted brain region and the frequency condition were set using NanoTherics Magnetherm to 7 mT and 595.4 kHz; a constant gradient field and alternating magnetic field was combined. A FFP was created at the center of the inner coil by concentrating the magnetic field with a gradient field.

Prior to the experiment, a gradient field was simulated by COMSOL to analyze the accuracy of targeting and the size of FFP and the heating efficiency of the MNPs. To verify focused heating, the temperature was measured using a sample holder with 5-mm-diameter holes. The holder consisted of 9 holes with a center-to-center distance of 1 cm. The increase in temperature at each location was recorded with an Osensa PRB-G40 probe; the overall temperature distribution was recorded with an Answer FLIR A65 camera. Based on a measured focused field for heating and temperature increases, a configuration was established for the in vivo experiments. Experimental setup of the permanent magnet and coil. A combined system was designed for the FFP and focused heating (Supplementary Fig. 4). Two disk-shaped permanent magnets were placed facing each other at a distance of 15.3 cm, and a coil was placed between the magnets. The head of the animal was placed inside the 5-cm-diameter coil. The head of a stroke model rat produced by the MCAO method was located at the center of the FFP with the customized conical head holder to fix the lesion at the center of the FFP. Animals who received stimulation treatment for 6 days were subjected to evaluations of functional recovery using the rotarod behavioral test and brain activation via PET/CT imaging.

### Temperature predication and estimation

Temperature setting for determining stimulation is one of the most important clinical factors. Therefore, the target temperature was set based on simulation results and ex vivo results for baseline brain temperature of animals. The temperature increasing in vivo was simulated using the SLP parameters representing the hyperthermia efficiency of the particles^35^. Predicted temperature based on simulation was validated with ex vivo temperature rise.

### Comsol simulation model

Heat distribution was analyzed using Comsol simulation for steady-state temperature rise induced by Fourier's law. At the nanoscale containing nanoparticles, the temperature changes without taking into account blood flow is expressed as follows^35,63^:

2 $$\Delta {\mathrm{T}}_{\mathrm{MNPs}}=\frac{\mathrm{SLP}\cdot {\mathrm{C}}_{\mathrm{MNPs}}\cdot {\left(6{\mathrm{V}}_{\mathrm{MF}}/\uppi \right)}^{2/3}}{8\cdot \mathrm{k}}$$

where V_MF_, C_MNPs_, and k are volume of magnetic fluid, concentration of MNPs, and thermal conductivity of biological tissues. The temperature increase in biological tissues is non-linear due to the homeostasis by blood flow and CSF, the rate of rising will be saturated. Therefore, a simplified brain-based in vivo heat transfer model was established to account for blood flow in living tissues. The bio-heat transfer equation for the brain model is expressed as follows^64–66^:

3 $${\uprho }_{\mathrm{br}}{\mathrm{C}}_{\mathrm{br}}\frac{\partial {\mathrm{T}}_{\mathrm{br}}}{\partial \mathrm{t}}={\mathrm{k}}_{\mathrm{br}}{\nabla }^{2}{\mathrm{T}}_{\mathrm{br}}+{\uprho }_{\mathrm{b}}{\mathrm{C}}_{\mathrm{b}}{\upomega }_{\mathrm{br}}({\mathrm{T}}_{\mathrm{b}}-{\mathrm{T}}_{\mathrm{br}})+{\mathrm{Q}}_{\mathrm{m}\_\mathrm{br}}+{\mathrm{Q}}_{\mathrm{MNPs}}$$

where ρ_br_ and ρ_b_ are densities of the brain and blood. C_br_ and C_b_ are specific heat of brain and blood. T_br_ and T_b_ are temperature of brain and arterial blood. k_br_, ω_br_, Q_m_br_, and Q_MNPs_ are the thermal conductivity of the brain, blood flow rate in brain, internal heat generation of the brain tissue and heat power density generated by the MNPs, respectively. Temperature and heat flow must be continuous on each interface between the brain/CSF, CSF/skull, skull/scalp, and scalp/air. The model with boundary conditions applied to each layer provides an environment similar to in vivo near the BBB. The predicted temperature rise values using particles of 15 mg/mL and 30 mg/mL concentrations were consistent with the experimental temperature rise values. Although there have been attempts to understand the physical theory of magnetic particle heat dissipation, it is beyond the scope of this study. Therefore, this study investigated functional improvement after the application of focused magnetic stimulation in vivo.

### Animal protocol

All animal procedures were performed in accordance with the approved protocol (GIST-2021-050) of the Institutional Animal Care and Use Committee within the Laboratory Animal Resource Center of the Gwangju Institute of Science and Technology, and performed in accordance with ARRIVE guidelines. All methods were performed in accordance with the relevant guidelines and regulations. Fifty specific pathogen-free naïve 8-week-old male Sprague–Dawley rats (300–400 g) were purchased. Each group was randomly allocated by a blinded investigator and then twenty-six rats were evaluated to confirm the change in BBB permeability after whole-brain and focused heating using Evans blue staining. Twenty-four rats were randomly separated into three groups to evaluate motor function recovery: control (MCAO, n = 6), sham (MNPs + focused heating, n = 9), and focused magnetic stimulation (MCAO + MNPs + focused magnetic heating, n = 9).

#### Modeling of MCAO

Various methods have been proposed to optimize the model for stroke mechanism research and clinical use. Modeling methods include a craniectomy model in which surgery is performed directly by exposing the MCA through cranial perforation^67,68^, a photothrombosis model that induces intravascular photo-oxidation by irradiating light at a specific wavelength^69,70^, and an endothelin-1 model that uses vasoconstrictive peptides^71,72^. However, the clinical applications of such models are limited because of surgical operations such as perforation of the skull, analysis problems involving endothelin-1 induced astrocytosis^73^, and mechanistic differences from human strokes^74^. Technically, the MCAO model is less invasive, and the technique used to occlude the MCA is most similar to the stroke mechanism in humans^75^.

Animals in the control and stimulation groups were subjected to MCAO stroke in the same manner as Koh et al.^76^. Rats were fixed on an operating table and anesthetized with 3% isoflurane. During surgery, body temperature was monitored with a rectal probe and maintained at 37 ± 0.5 °C with a warming plate. A 3-cm longitudinal incision was made to expose the left common carotid artery (CCA), left external carotid artery (ECA), and left ICA. A nylon thread pre-coated with silicon resin was inserted into the left ICA to occlude the MCA intersection. After 100 min of ischemia, the tied thread was removed and reperfusion of the ischemic area was performed via the left CCA. After surgery, all animals were returned to their cages without behavioral restrictions.

Step-by-step experiment of the animal protocol was performed (Supplementary Fig. 5). A stroke is considered to be in the chronic phase when neurological recovery is complete and behavioral function becomes constant after a certain period of acute stroke^40,77,78^. Rodent models of stroke are generally considered to be in the chronic phase at 4 weeks after stroke induction^78–82^. The rats subjected to stroke were randomly separated into a control group and an experimental group for the rotarod behavioral test 4 weeks after stroke induction.

#### Focused magnetic stimulation group

A chronic stroke model was prepared as described above to apply focused magnetic heating. The stimulation was conducted for 6 days; neurologic deficit score (NDS) analysis and PET/CT images were obtained on the first day and the last day. The rotarod test was conducted on the first and last day of the experiment. PET/CT images were acquired after each rotarod test. On day 2, after an incision had been made in the same manner described for stroke modeling, the left CCA and ECA were temporarily tied by silk suture. A 24-gauge catheter was applied from the CCA to an inlet of the ICA; 60 µL of MNPs at 15 mg/mL were then slowly injected into the ICA. After injection, bleeding from vessels was stopped using the surrounding tissue and the tied suture was removed for reperfusion.

Focused magnetic heating was applied immediately after particle injection; the field was applied at the same time each day for 30 min under anesthesia with 2% isoflurane. The head of the animal was placed in a magnetic field inside the coil and fixed in a customized conical holder, such that the striatum region was exposed to the focal field created by the gradient field. MNPs were injected once on days 2 and 5; focused magnetic heating was applied for 6 days. On day 8, rotarod behavioral assessment and PET/CT imaging were performed to evaluate the effects of hyperthermia.

#### Sham group

Animals in the sham group did not undergo MCAO surgery; they were isolated for the same period as the magnetic stimulation group, and the rotarod test and PET/CT imaging were performed as discussed above. Naïve rats were anesthetized with 3% isoflurane in oxygen and placed on the plate. Animals in this group were anesthetized for the same number of times and duration as in the magnetic stimulation group. After the neck skin incision had been made, the same MNP dose was injected into the left ICA in the same manner as in the magnetic stimulation group. After the nanoparticles had been injected, the animals were exposed to focused magnetic heating for 6 days same as magnetic stimulation group; the rotarod behavior test and PET/CT imaging were then performed.

#### Control group

Animals in this group were isolated for 8 days at 4 weeks after MCAO surgery; they were not exposed to magnetic fields. Rotarod behavioral assessment and PET/CT imaging were performed without hyperthermia.

### Evans blue staining and fluorescence image for reversible changes in BBB permeability

Magnetic heating-induced changes in BBB permeability were analyzed using Evans blue staining. BBB permeability begins to increase from 38 to 39 °C^37^. However, direct measurement of the temperature near the BBB using a thermometer is impossible; the temperature was assessed by quantitative evaluation of dye spread in the tissue^27^. MNPs were injected into the ICA as previously described to confirm magnetic stimulation-induced changes in BBB permeability. Whole-brain and focused magnetic heating were applied to the rats in which MNPs had spread throughout the brain. After the injection of 30 mg/mL MNPs and the application of stimulation, 1 mL of 4% Evans blue was injected through the tail vein to assess BBB permeability immediately after heating, after 24 h, and after 48 h. After 2 h of Evans blue circulation, animals were sacrificed by intracardiac perfusion of saline to flush blood and Evans blue dye out of the vasculature. Evans blue staining was imaged with fluorescence analysis after brain extraction.

The extracted brain was sliced into 2-mm-thick coronal sections; vascular permeability was evaluated by measuring the extravasation of Evans blue using in vivo imaging system (IVIS lumina S5, PerkinElmer). Ex vivo brain images were acquired of both the whole-brain and 2-mm-thick tissue slices with the following parameters: excitation filter, 600 nm; emission filter, 710 nm; exposure time, 1 s; binning, 8; f-stop, 2; and field of view, 21 cm. Whole-brain and brain-slice region of interest (ROI) were analyzed by observing Evans blue fluorescence. Relative fluorescence values after stimulation were calculated within the ROI, the brain stimulation region. The degree of Evans blue leakage between groups was analyzed by quantifying the total amount of Pixel Intensity.

### Behavioral test for functional evaluation and neurologic deficit score

The functional outcome of the animal model, along with histological measurements, provides insight into stroke mechanisms and potential rehabilitation. An assessment sensitive to the detection of various impairments, ranging from global to specific modalities, after stroke allows evaluation of the functional outcomes after stroke in rodent models^83^. Because ischemic animals have more neurological deficits than do non-ischemic animals, scores were evaluated on four scales including gait and limb tone^84–86^. A neurological deficit that may occur with heat was examined with designated tests before and after stimulation. An investigator blinded to the all groups determined the NDS based on (a) consciousness: normal, 0; restless, 1; lethargic, 2; stuporous, 3; (b) gait: normal, 0; paw adduction, 1; unbalanced walking, 2; circling, 3; unable to stand, 4; no movement, 5; (c) limb tone: normal, 0; spastic, 1; flaccid, 2; and (d) pain reflex: normal, 0; hypoactive, 2; absent, 4. A higher NDS indicated a worse neurological status.

The rotarod test was used to evaluate motor deficits and balance alteration caused by brain injury in rodents^87–89^. The rotarod apparatus records the time that the animal remains on the device; ischemic animals generally spend less time on the device^87,90^. To verify motor function recovery, motor impairment was evaluated using the accelerated rotarod test before and after stimulation. A 5-min activity test was performed for each group using Med-associates ENV-577. The rat was placed on the rod, which accelerated at 20 rpm/min until 8 rpm was reached. The rat would then grab the rod and begin walking forward. The rod was rotated for up to 5 min until the rat fell; the time required for the rat to fall off the rod was recorded. Three consecutive trials were conducted for each session, and the average time to fall was calculated after three trials. Effects within the focused magnetic stimulation group were analyzed using the nonparametric Wilcoxon signed rank test, which compared pairs of two-level matched samples before and after heating.

### Brain activation imaging

In PET imaging, an isotope was injected into a vein and distributed in different tissues depending on the carrier. A positron-emitting radiopharmaceutical ^18^F-fluorodeoxyglucose (FDG) was injected for preclinical studies of brain glucose metabolism; its origin was estimated by back-projecting photons emitted by the interaction between the free electron and positron from the isotope. A localized signal from photons was used to form a slice by means of a reconstruction algorithm. PET images were fused with computed tomography images for the anatomical localization of radiopharmaceuticals^91^.

In this study, glucose metabolism at a brain site was evaluated in vivo using a commercial micro-PET scanner (Concorde Microsystems, Knoxville, TN, USA) before and after focused magnetic stimulation. After intravenous injection of 2 mCi/mmol ^18^F-FDG through the tail vein to rats under halothane gas anesthesia, each brain-slice image was acquired on a micro-PET scanner 60 min later. The collected data were reconstructed with a maximum posterior probability algorithm using a pixel size of 0.4 × 0.4 × 1.2 mm^3^, then displayed and analyzed by IDL (Research Systems, Boulder, CO, USA). Glucose metabolism was analyzed by plotting ROIs in the cerebrum, cortex, and subcortex for each slice. A semi-quantitative SUV method was used to calculate metabolic changes in both the cerebral cortex and subcortex areas.

PET images were analyzed using PMOD (v4.1, PMOD Technologies, Switzerland). For quantitative analysis, each PET image was transformed into the rat brain template (W. Schiffer) via manual co-registration. After application of the brain VOI atlas, quantitative values of cerebral glucose uptake were derived in each sub-region of the rat brain. To compare PET signal intensity values between the first and second scan in the same rat, glucose uptake activity (%ID/g) was determined. To create a subtraction image for display, processing steps were included to remove excess signal from the neck and sides of the head, register the first and second scans to the atlas, normalize intensity via proportional scaling, and subtract the first scan from the second scan. The difference between the first and second scan was calculated as the uptake ratio (%), defined as follows: (mean of second scan − mean of first scan)/mean of first scan × 100^92^. Statistics of SUV indices within ROI were analyzed by the nonparametric Mann–Whitney U test.

## Supplementary Information

Supplementary Figures.

## Data Availability

All data generated or analyzed during this study are included in the main text.
