# Supplementary material for: Magnetothermal-based non-invasive focused magnetic stimulation for functional recovery in chronic stroke treatment
Source: Sci Rep. 2023 Mar 27;13:4988. doi: 10.1038/s41598-023-31979-w (PMC10042827; doi:10.1038/s41598-023-31979-w)
Supplement: Supplementary file 1 — Supplementary Figures. [file 41598_2023_31979_MOESM1_ESM.docx]

***Supplementary Materials***


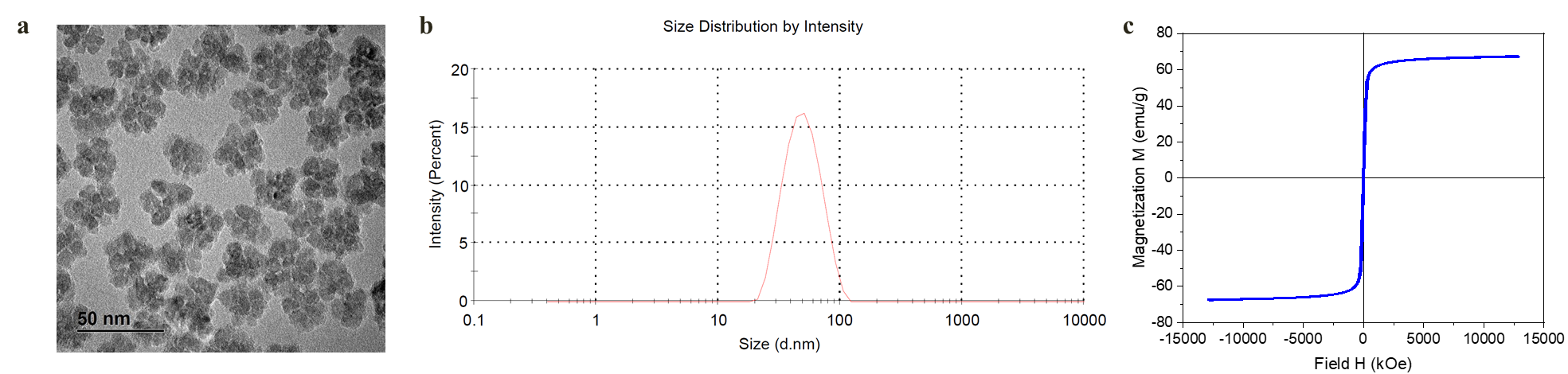


**Figure 1. Physical properties of MNPs.** (**a**) TEM revealed a core-size distribution of 25 nm $\pm$ 3 nm. (**b**) Hydrodynamic size consisted of an average diameter of 45 nm $\pm$ 15 nm and a peak size of 50.5 nm. (**c**) Magnetization was measured for suitability for *in vivo* experiments according to saturation magnetization. VSM imaging showed a peak saturation magnetization value of 67 emu/g, which can generate high SLP *in vivo* experiments.


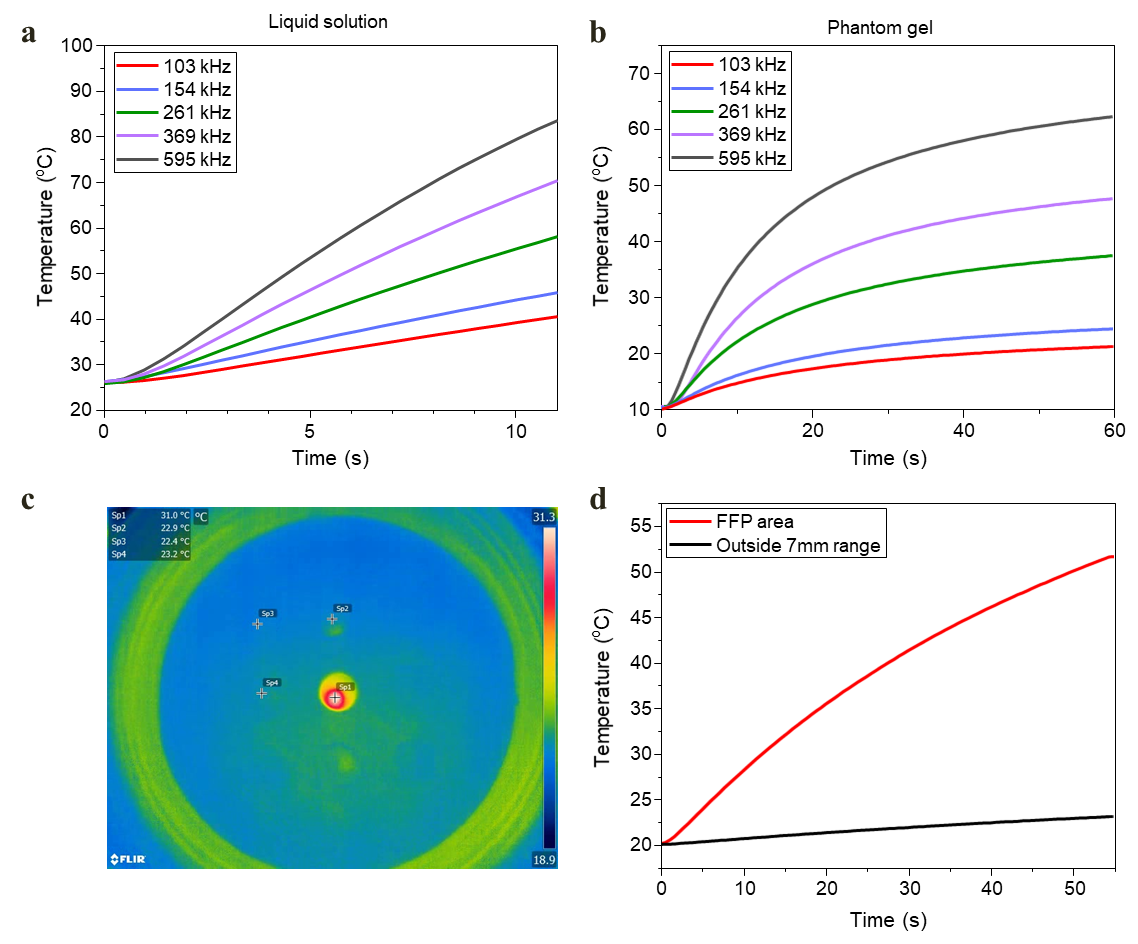


**Figure 2. Particle heating conditions and target temperature.** 100 μL of 30 mg/mL MNPs showed high heating efficiency at high frequency in liquid solution (**a**) and also in phantom gel to eliminate Brownian motion (**b**). The temperature of each sample shown in (**c**) distributed inside the phantom holder was measured using an infrared camera. The temperature significantly increased in the central region where the FFP is present; the rest of the outer position remained at room temperature. Based on a measured focused field for heating and temperature increases, a configuration was established for the in vivo experiments. (**d**) A temperature increase of 100 μL of 30 mg/mL MNPs under focused magnetic heating inside the phantom holder. The exact temperature was compared by direct measurement using a temperature probe. A significant increase in temperature was observed in the center.


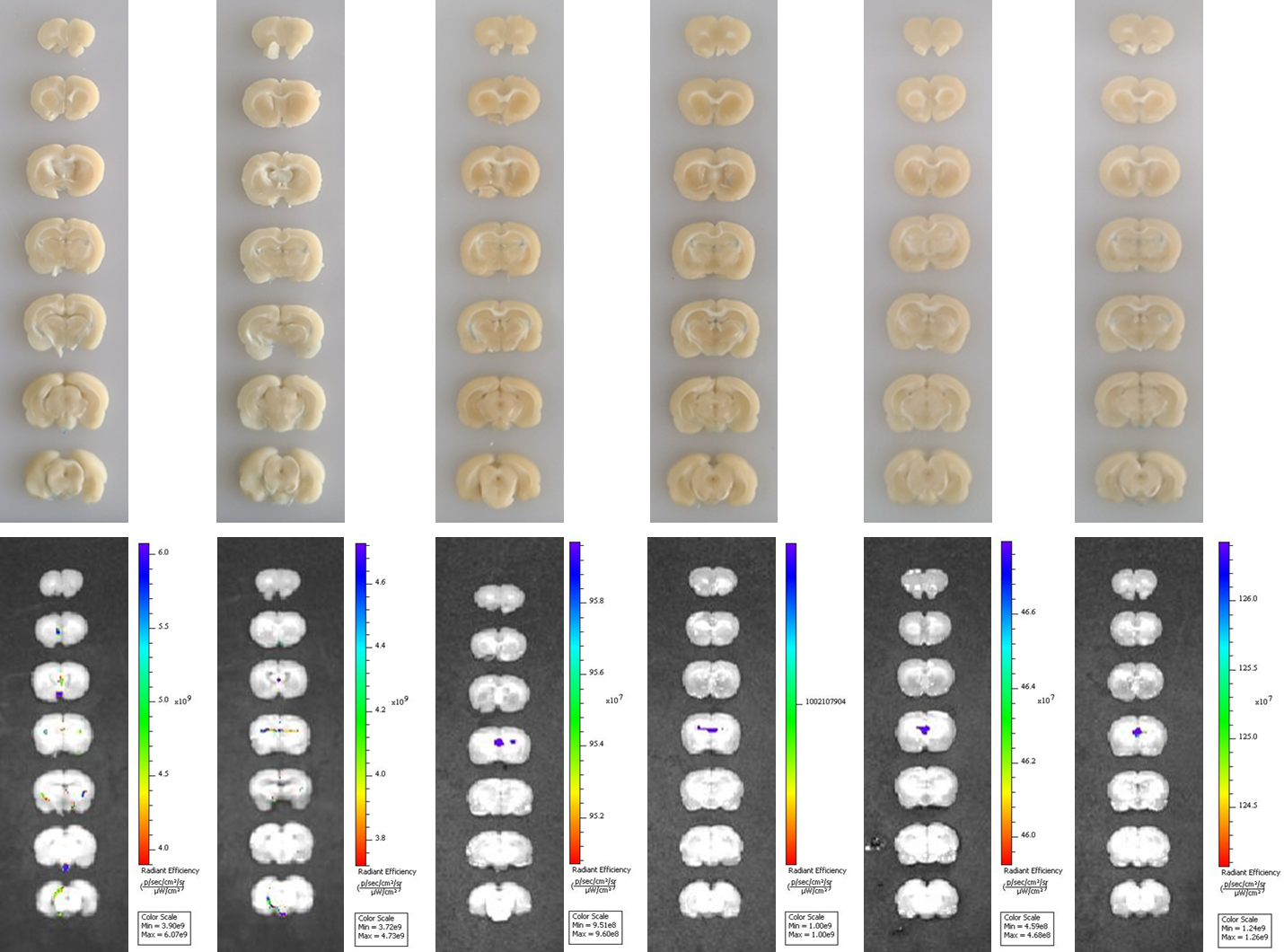


**Figure 3. IVIS images of the sham group after focused magnetic stimulation.** After the application of focused magnetic stimulation, no damage to the BBB was observed through brain slices and Evans blue staining of each rat.


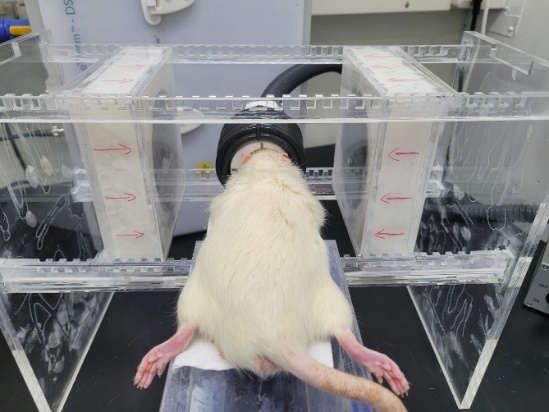


**Figure 4. Setup for focused magnetic heating *in vivo*.** A 7 mT magnetic field at 595.4 kHz was applied under a gradient field of 2.1 T/m.


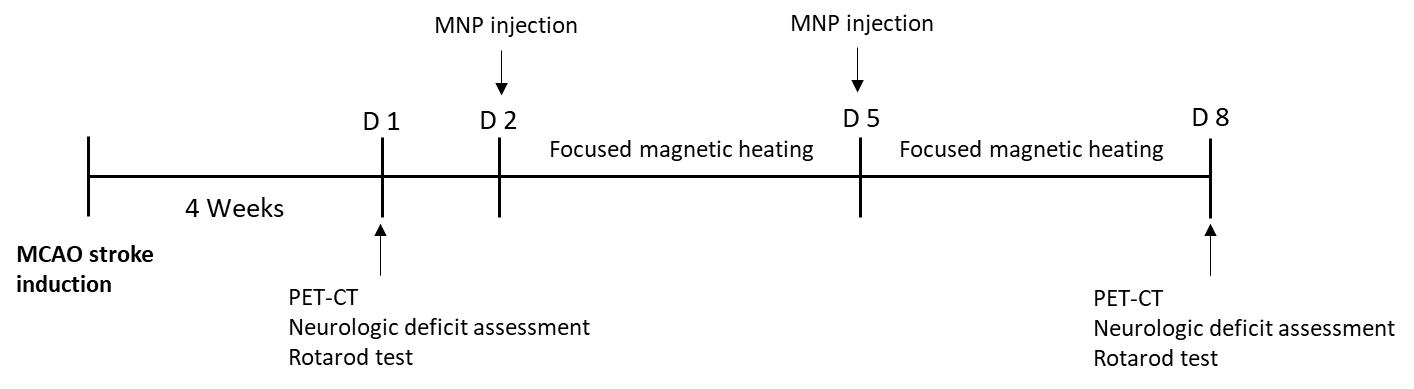


**Figure 5.** **Timeline of focused magnetic stimulation.** After 4 weeks of chronic-stroke model preparation, focused magnetic hyperthermia was applied for 6 days. PET images and behavioral tests were performed before and after heating.
